# Anxiety and depression lowers blood pressure: 22-year follow-up of the population based HUNT study, Norway

**DOI:** 10.1186/1471-2458-11-601

**Published:** 2011-07-28

**Authors:** Bjørn Hildrum, Ulla Romild, Jostein Holmen

**Affiliations:** 1 Department of Psychiatry, Namsos Hospital, Nord-Trøndelag Health Trust, Namsos, Norway; 2 Department of Research and Development, Namsos Hospital, Nord-Trøndelag Health Trust, Namsos, Norway; 3 Swedish National Institute of Public Health, Östersund, Sweden; 4 HUNT Research Centre, Faculty of Medicine, Norwegian University of Science and Technology, Levanger, Norway

## Abstract

**Background:**

For decades, symptoms of anxiety and depression have been included among psychological factors associated with development of hypertension. Although this has been questioned in recent studies, most findings have been based on a single assessment of mental distress at baseline. We examined these associations using repeated assessments of anxiety, depression and blood pressure.

**Methods:**

Data on 17,410 men and women aged 20 to 67 participating in the Nord-Trøndelag Health Study (HUNT) in Norway in 1984-86 were re-examined 11 and 22 years later. The main outcome was change in mean blood pressure (mm Hg) during follow-up.

**Results:**

We found that a high symptom level score (≥80^th ^percentile) of combined anxiety and depression at baseline, as compared to a lower symptom level, was associated with lower mean systolic (-0.67 mm Hg, p *= *0.044) and diastolic (-0.25 mm Hg, p *= *0.201) blood pressure at year 22. A high symptom level present at all three examinations was associated with a stronger decrease in mean systolic (-1.59 mm Hg, p *= *0.004) and diastolic (-0.78 mm Hg, p *= *0.019) blood pressure and with a 20% (p = 0.001) lower risk of developing hypertension (BP ≥140/90 mm Hg) at year 22. The associations were only slightly attenuated in multivariate analyses, with no evidence of a mediating effect of alteration in heart rate.

**Conclusions:**

This study do not support previous hypothesis that emotional stress may be a cause of hypertension. Our findings indicate that symptoms of anxiety and depression are associated with decrease in blood pressure, particularly when a high symptom level can be detected over decades.

## Background

The origin of the hypothesis that emotional stress might be a cause of hypertension goes back to 1919 when Moschcowitz described features of a stressed personality type in hypertensive patients [1]. The hypothesis was later moderately supported in several studies, with the strongest support for anger and for symptoms of anxiety and depression leading to hypertension; however, the psychometric properties of the applied scales are criticized [2]. In recent years, population studies have not consistently reproduced previous hypothesis and results. On the contrary, depression and anxiety, the two major psychiatric disorders, were in several studies associated with lower blood pressure [3-10]. This was also the case in the largest general population-based study to date where we found that symptoms of anxiety and depression predicted lower blood pressure 11 years later [11]. In contrast, a recent study on a cohort of civil servants using repeated measures of depressive symptoms and blood pressure over 24 years suggested that the risk of hypertension increased with repeated depressive episodes [12]. Four other prospective studies with non significant or contradictory findings were hard to interpret due the use of self-reported hypertension [13-15], positive findings in middle-aged but not in elderly individuals [13], or a short period of follow-up [16].

Overall, previous studies in this area differ in their findings. With a few exceptions [12,17], however, they have based their analyses upon a single assessment of mental distress at baseline. Therefore, in the present study we wanted to extend our previous analyses [11] by using repeated measurements (baseline, year 11 and year 22) of anxiety and depression symptoms and of blood pressure within the same community-based population. The main aim was to study the effect of high level of combined anxiety and depression on blood pressure through 22 years; secondly, we wanted to study the individual effects of anxiety only and of depression only on blood pressure; and thirdly, whether alteration in heart rate could explain these associations.

## Methods

### Participants

This study is part of the Nord-Trøndelag Health Study (the HUNT Study, http://www.hunt.no), Norway. All inhabitants in the county aged ≥20 years were invited to a general health study that was conducted three times with 11-year intervals: in 1984-86 (HUNT 1 = baseline) [18], 1995-97 (HUNT 2 = year 11) [19], and 2006-08 (HUNT 3 = year 22). Data were obtained from physical examinations, blood samples, and from questionnaires that covered demographic characteristics, somatic illnesses, somatic and mental symptoms, medication, life style, and health-related behaviour.

In the present 22-year longitudinal study we included individuals aged 20-67 years at baseline (HUNT 1) who participated in all three surveys with valid data on anxiety/depression, blood pressure, heart rate and waist circumference. Of the 48,907 HUNT 1 participants with valid data, 5,990 died or moved out of the county during the first 11 years of follow-up. Among the remaining 42,917 individuals, 34,263 (79.8%) participated with valid data in HUNT 2. Of these individuals, 4,725 died or moved out of the county during the next 11 years of follow up. Of the remaining 29,538 individuals, 58.9% participated in HUNT-3 with valid data, giving a study population of 17,410 participants (7888 men, 9522 women) aged 20-67 years at baseline (42-89 years at year 22). Compared with participants (n = 17,410), those who did not complete the study (n = 12,128) among eligible individuals at year 22 were older (53 versus 51 years) and were less often women (52.5 versus 54.7%), had slightly higher mean blood pressure (140.5/82.3 versus 136.5/81.5 mm Hg) and higher symptom scores of anxiety (4.33 versus 4.20) and depression (3.76 versus 3.50); p ≤ 0.001 for all comparisons. However, we found no interaction between age or sex and the association between anxiety/depression and blood pressure [6,11]. Therefore, we did not expect non-completers to bias our findings. The personal identification number allowed linkage of data for each individual. The Norwegian Data Inspectorate and The Regional Committee for Ethics in Medical Research approved the HUNT study and the present follow-up study.

### Anxiety and depression

Twelve questions on anxiety and depression were included in the baseline questionnaire, constituting a one-dimensional anxiety and depression symptom index (the ADI-12 Index) which was found to correlate strongly (r = 0.82) with the Hopkins Symptoms Checklist-25 [11]. The Hospital Anxiety and Depression Scale (HADS) was employed at year 11 and at year 22. The HADS is a self-report questionnaire comprising of 14 four-point Likert-scaled items, seven for anxiety (HADS-A) and seven for depression (HADS-D) [20].

We previously reported that the cross-sectional associations between a *combined anxiety-depression score *and blood pressure were almost identical at baseline and at 11-year follow-up, despite use of different measurements of anxiety and depression and of blood pressure [11]. Cross-sectional analyses at 11-year follow-up also indicated that HADS-defined anxiety and depression, respectively, were similarly associated with blood pressure [6]. Consequently, for the 22-year longitudinal analyses, one-dimensional measures for symptoms of anxiety and depression were calculated at baseline, 11-year and 22-year follow-up. Using a categorical approach, we defined a high symptom level of anxiety/depression at all three measurements as scores above the 80^th ^percentile on the ADI-12 Index at baseline and as combined HADS-A and HADS-D scores ≥12 (equals around the 80^th ^percentiles) at 11-year and at 22-year follow-up. Finally, we used continuous measures of *anxiety *(HADS-A) and of *depression *(HADS-D), respectively, at 11-year follow-up in analyses of their individual effects on change in blood pressure from 11-year to 22-year follow-up. Supplementary analyses were performed using well-established anxiety/depression cut-offs (HADS-A ≥ 8, HADS-D ≥ 8) [20].

### Blood pressure and heart rate

Specially trained nurses measured blood pressure in seated participants, after four minutes (baseline) or two minutes (years 11 and 22), with the cuff placed on the right upper arm, and with the arm rested on a table at heart-level. Cuff size was adjusted after measuring the arm circumference. At baseline, blood pressure was measured using calibrated mercury manometers with standard cuff size. The first pulse sound (Korotkoff's phase I) was registered as systolic blood pressure and the level at which the pulse disappeared (phase V) as diastolic blood pressure. The measurements were repeated after two minutes, and the second reading was used in this study. At years 11 and 22, blood pressure was measured with a Dinamap 845XT (Criticon, Florida, USA) based on oscillometry. Blood pressure was measured automatically three times at one-minute intervals. The mean of the second and third reading was used in this study. At HUNT 1, resting heart rate at the wrist was counted for 15 seconds or for 30 seconds if the heart was irregular. At HUNT 2 and 3, heart rate was measured by the Dinamap. Heart rate was expressed as beats/min.

### Outcome measures

The main outcome measures were change in mean systolic or diastolic blood pressure (mm Hg) from baseline to year 22, and from year 11 to year 22. In logistic regression analyses we used blood pressure ≥ 140/90 mm Hg or < 120/75 mm Hg at year 22 as the outcome measures.

### Statistical analysis

Characteristics of the participants were calculated as means (± standard deviation) or percentages. We used linear regression models to analyse the association of *combined anxiety and depression *with change in blood pressure from baseline to year 22. Symptoms of anxiety and depression were first included as a continuous measure at baseline, and then categorically as a high symptom level at baseline, at both baseline and year 11, and at all three measurements, respectively. Effects were reported as unstandardised regression coefficients (b = mm Hg change in blood pressure during follow-up). We repeated analyses after excluding those taking antihypertensive or antidepressant medication, respectively.

We used three regression models. First, we analysed the association with adjustment for demographic factors (age, sex, educational level) and systolic or diastolic blood pressure at baseline. Second, we further adjusted for angina pectoris, myocardial infarction, stroke, diabetes mellitus, smoking status, low physical activity, antihypertensive medication (baseline and change during follow-up, all categorical); hypertension among siblings (baseline and change from baseline to year 11); and waist circumference (year 11 and change from year 11 to year 22, both continuous). Third, after examining separately the associations of heart rate with anxiety and depression and with blood pressure, we included adjustment for heart rate (baseline and change during follow-up, both continuous) in the analyses of the association of anxiety and depression with blood pressure.

We also analysed the individual effects of *anxiety *and of *depression*, respectively, at year 11 on change in blood pressure from year 11 to year 22. In a second model, we examined the effects of pure anxiety and of pure depression by adjusting for each other.

In additional logistic regression analyses we examined the effect of a high symptom level of anxiety/depression at all three measurements on blood pressure at year 22 dichotomised into hypertension (≥ 140/90 mm Hg) or not, and then with blood pressure dichotomised into "hypotension" (< 120/75 mm Hg) [4,10] or not, with adjustment for age, sex and educational level.

Data were analysed using SPSS version 18.0 for Windows (SPSS Inc, Chicago, Illinois, USA).

## Results

Characteristics of the sample are listed in Table 1. An increase in blood pressure the first 11 years was followed by a decrease the next 11 years. Systolic blood pressure at baseline correlated strongly with that of year 11 (*r *= 0.61) but more weakly with that of year 22 (*r *= 0.38). For diastolic blood pressure, the corresponding figures were *r *= 0.52 and *r *= 0.26. The correlations between the combined anxiety and depression score at baseline (the ADI-12 Index) and the HADS at years 11 and 22 were *r *= 0.45 and *r *= 0.42, respectively. The correlation between HADS-A at years 11 and 22 was *r *= 0.59. For HADS-D, the correlation was *r *= 0.55. (p < 0.001 for all correlations). There were only minor variations in the levels of HADS-A and HADS-D scores at years 11 and 22.

**Table 1:** Characteristics of participants (n = 17,410) at baseline, at 11-year, and at 22-year follow-up

	Baseline (HUNT 1, 1984-86)	11-year follow-up (HUNT 2, 1995-97)	22-year follow-up (HUNT 3, 2006-08)
Demographics			
Age, y	40.0 ± 10.6		
Sex, % female	54.7		
Educational level, %			
Low	37.3		
Medium	48.6		
High	14.0		
HADS^1 ^defined symptom level			
Anxiety		4.2 ± 3.2	3.9 ± 3.2
Depression		3.5 ± 2.9	3.6 ± 2.9
Blood pressure, mm Hg			
Systolic	126.7 ± 15.9	136.5 ± 19.3	135.1 ± 19.3
Diastolic	80.9 ± 10.5	81.5 ± 11.2	75.2 ± 11.1
Heart rate, bpm	73.0 ± 11.6	71.6 ± 12.2	69.4 ± 11.7
Medical history, %			
Angina pectoris	0.7	2.7	5.1
Myocardial infarction	0.3	1.8	4.6
Stroke	0.2	0.8	3.4
Diabetes mellitus	0.5	1.7	5.7
Hypertension among siblings	9.9	11.8	No data
Lifestyle and health factors			
Waist circumference, cm	No data	86.0 ± 10.9	95.1 ± 11.6
Low physical activity, %^2^	39.4	26.1	21.9
Smoking, %	31.0	25.6	17.8
Medication			
Antidepressants, %	No data	3.2	No data
Antihypertensives, %^3^	4.7	11.5	29.0

Data are percentages or means ± standard deviation.

Bpm, beats per minute

^1^The Hospital Anxiety and Depression Scale (At HUNT 1, a one-dimensional anxiety and depression symptom index was used).

^2^Defined as physical activity less than once a week (HUNT 1 and 3) or less than one hour a week (HUNT 2).

^3^Previously or currently.

### Effect of combined anxiety and depression on mean blood pressure

We found symptoms of anxiety and depression to predict a relative decrease in blood pressure during 22-year follow-up. Baseline level of anxiety and depression was positively associated with a decrease in systolic blood pressure in linear regression analyses adjusted for age, sex, educational level and baseline blood pressure. For diastolic blood pressure, we found a similar non-significant trend. A high symptom level at both baseline and at year 11 was more strongly associated with a decrease in blood pressure during 22-year follow up. In individuals with a high symptom level at all three examinations, we found an even stronger decrease for both systolic and diastolic blood pressure (b = -1.59, p = 0.004, and b = -0.78, p = 0.019, respectively) compared with individuals with a lower symptom level. Additional adjustment for other health factors and for antihypertensive medication did not change the overall pattern of results, but the effect of combined anxiety and depression on blood pressure was slightly attenuated (Table 2).

**Table 2:** Association of combined anxiety/depression scores^1 ^with change in blood pressure from baseline (HUNT 1) to 22-year follow-up (HUNT 3)

	**Adjusted for age, sex, educational level, and baseline BP**^**2 **^			**Additionally adjusted for health factors and medication**^**3**^			**Additionally adjusted for heart rate**^**4**^		
	**B**	***P***	***R***^***2***^	**B**	***P***	***R***^***2***^	**B**	***P***	***R***^***2***^
Combined anxiety/depression	***Systolic blood pressure***								
Baseline, per SD increase	-0.39	0.009	0.22	-0.32	0.032	0.23	-0.33	0.025	0.24
High at baseline	-0.67	0.044	0.22	-0.55	0.099	0.23	-0.61	0.067	0.24
High at baseline and at 11 y	-1.27	0.006	0.22	-1.15	0.012	0.23	-1.21	0.008	0.24
High at baseline, 11 y and 22 y	-1.59	0.004	0.22	-1.49	0.007	0.23	-1.51	0.006	0.24
									
Combined anxiety/depression	***Diastolic blood pressure***								
Baseline, per SD increase	-0.17	0.054	0.38	-0.12	0.176	0.39	-0.13	0.145	0.42
High at baseline	-0.25	0.201	0.38	-0.15	0.439	0.39	-0.21	0.278	0.42
High at baseline and at 11 y	-0.54	0.050	0.38	-0.44	0.110	0.39	-0.51	0.058	0.42
High at baseline, 11 y and 22 y	-0.78	0.019	0.38	-0.73	0.026	0.39	-0.75	0.019	0.42

BP, blood pressure; SD, standard deviation; B, mm Hg change in BP during follow-up, from linear regression analyses.

^1^Estimated with the Anxiety and Depression Symptom Index-12 (HUNT 1) and the Hospital Anxiety and Depression Scale (HUNT 2 and 3). A high symptom level was defined as score above the 80^th ^percentile of the two scales.

^2^Systolic or diastolic.

^3^Angina pectoris, myocardial infarction, stroke, diabetes mellitus, smoking status, low physical activity, antihypertensive medication (baseline and change during follow-up); hypertension among siblings (baseline and change from baseline to year 11); and waist circumference (year 11 and change from year 11 to year 22).

^4^Baseline and change during follow-up.

Excluding individuals using antidepressant medication at year 11 (remaining: n = 16,852) did not change the results essentially: a high symptom level at all three examinations was associated with a slightly lower decrease in systolic (b = -1.30, p = 0.023) and diastolic (b = -0.66, p = 0.047) blood pressure. However, after excluding those using antihypertensive medication at baseline or at year 22 (remaining: n = 12,235), we found a stronger effect on systolic (b = -2.51, p < 0.001) and diastolic (b = -1.26, p = 0.001) blood pressure.

### Effect of pure anxiety and pure depression on mean blood pressure

In analyses using symptoms of anxiety and depression as continuous scores separately at year 11, we found that both anxiety and depression predicted lower blood pressure at year 22. In analyses where anxiety and depression were further adjusted for each other, the remaining (pure) effects of anxiety and of depression on blood pressure were attenuated or became non-significant (Table 3). Further adjustment for other health factors, antihypertensive medication and heart rate slightly attenuated the associations, similarly as shown in Table 2. Supplementary analyses using anxiety/depression cut-offs (HADS-A ≥ 8, HADS-D ≥ 8) confirmed the direction and increased the strength of the associations described above. However, several of the findings became non-significant (Table 3).

**Table 3:** Association of anxiety and depression^1 ^at 11-year follow-up (HUNT 2) with change in blood pressure from 11-year to 22-year follow-up (HUNT 3)

	**Adjusted for age, sex, educational level, and BP**^**2 **^**at HUNT 2**			Additionally adjusted for depression or anxiety, respectively		
	**B**	**P**	**R**^**2**^	**B**	**P**	**R**^**2**^
**Systolic blood pressure**						
Anxiety, per SD increase	-0.41	0.001	0.26	-0.23	0.145	0.26
Anxiety, case level^3^	-0.70	0.049	0.26	-0.54	0.159	0.26
Depression, per SD increase	-0.46	< 0.001	0.26	-0.31	0.045	0.26
Depression, case level^3^	-0.74	0.078	0.26	0.48	0.294	0.26
**Diastolic blood pressure**						
Anxiety, per SD increase	-0.21	0.004	0.34	-0.15	0.102	0.34
Anxiety, case level^3^	-0.40	0.051	0.34	-0.31	0.161	0.34
Depression, per SD increase	-0.20	0.006	0.34	-0.11	0.223	0.34
Depression, case level^3^	-0.42	0.085	0.34	-0.27	0.303	0.34

BP, blood pressure; SD, standard deviation; B, mm Hg change in BP during follow-up from linear regression analyses.

^1^Measured with the Hospital Anxiety and Depression Scale (HADS).

^2^Systolic or diastolic.

^3^Case level = score ≥ 8 on HADS-A or HADS-D, respectively.

### Heart rate

At baseline, an increase in symptoms of combined anxiety and depression (per standard deviation) was associated with a dose-response increase in heart rate, as shown by regression coefficient (b) = 0.36, p < 0.001, in cross-sectional analyses adjusted for age, sex and educational level. Prospectively, a high symptom level at all three examinations was non-significantly associated with an increased heart rate at year 22 (b = 0.52, p = 0.16) compared with individuals with a lower symptom level. Similarly, at baseline, an increase in heart rate was associated with an increase in systolic (b = 0.28, p < 0.001) and diastolic (b = 0.14, p < 0.001) blood pressure. Prospectively, an increase in heart rate from baseline to year 22 was associated with an increase in systolic (b = 0.16, p < 0.001) and diastolic (b = 0.14, p < 0.001) blood pressure.

Including additional adjustment for heart rate in the analyses of anxiety and depression with change in blood pressure improved the models, as shown by an increase in explained variance (adjusted R^2^). In all analyses, we found that additional adjustment for heart rate attenuated the adjusting effect of the other covariates (Table 2).

### Effect of combined anxiety and depression on hypertension or "hypotension"

Logistic regression analyses confirmed the results from the linear regression analyses. We found that a high symptom level of combined anxiety/depression at all three measurements, compared to a lower symptom level, was associated with reduced odds for blood pressure ≥ 140/90 mm Hg at year 22 (odds ratio 0.80, confidence interval 0.70 - 0.92) and, conversely, with higher odds for blood pressure < 120/75 mm Hg (odds ratio 1.20, confidence interval 1.05 - 1.36). Thus, the probability for development of hypertension among individuals with a high symptom level of anxiety/depression at all three examinations was 20% lower compared to those with a lower symptom level.

## Discussion

Recently, we reported that symptoms of combined anxiety and depression predicted a decrease in blood pressure 11 years later in both sexes and in all adult age groups [11]. The present study extends our previous findings by showing that the decrease in blood pressure was even stronger when a high symptom level was present at all three examinations (baseline, year 11 and year 22). We also found that both anxiety and depression separately contributed to the lowering of blood pressure. Furthermore, the associations were only slightly attenuated in multivariate analyses, with no evidence of a mediating effect of alteration in heart rate.

Although one might question the clinical value of a modest reduction in mean blood pressure, studies have suggested that this might translate into significant public health benefits [21]. In our population we found that a high symptom level of anxiety and depression at all three examinations reduced the risk of being hypertensive at year 22 by 20%.

The study confirms and extends findings from other large population-based studies indicating that symptoms of anxiety and depression predict lower blood pressure [3-11]. Together, these findings contrast with the hypothesis that the well established association between depression and cardiovascular disease might be explained by elevated blood pressure [22]. In a previous study, we have discussed conflicting results in other reports [11]. Recently, four studies with non significant or contradictory findings were hard to interpret due the use of self-reported hypertension instead of direct measurement of blood pressure [13-15], significant findings in middle-aged but not in elderly [13], a short period of follow-up [16], and a possible mediating role of tricyclic antidepressants [16].

Our findings also contrast with a recent study based on data from the British Whitehall II Study suggesting that the risk of hypertension increased with repeated experience of depressive symptoms over time [12]. However, although this study benefited from five waves of screening data of depressive symptoms and blood pressure, it had some limitations. First, it included only civil servants aged 35 to 55 years at baseline; thus, it was not representative of the general population. Second, the findings were not significant in women. Third, covariates (such as smoking, physical activity, body mass index and medical conditions) were only assessed at baseline. These limitations may perhaps explain some of the inconsistency between this study [12] and our study. In addition, compared to the HUNT study, the Whitehall study had a much higher proportion (11%) of non-white participants in which they reported higher odds for hypertension. Of note, in the present study we also included anxiety; analysed mental symptoms and blood pressure both as continuous and as categorical variables; and adjusted for change in covariates (including heart rate) during follow-up.

With a few exceptions [12,17], previous studies on this topic have assessed mental distress only at baseline. We examined these associations in a large community-based population using repeated assessments of anxiety, depression and blood pressure. We found that the effect of anxiety and depression on blood pressure was more clearly uncovered with increasing number of assessments, which increase the reliability of measurements as compared to a single one. Moreover, it seems reasonable that ongoing symptoms of anxiety and/or depression have stronger long-term physiological effects than a shorter period of mental distress. Therefore, the authors of a previous study emphasized the importance of repeated assessments when evaluating the effect of mental distress on blood pressure [17]. They reported partly opposite findings as compared to ours; however, their study had a small sample size prone to selection bias.

Blood pressure, heart rate, and depression are all related to cardiovascular mortality, whereas the literature on anxiety is more conflicting [23]. Regarding heart rate, a high level seems to be a marker of cardiovascular events in general populations [24] and in patients with established cardiovascular disease [25]. It is also reported to be associated with depressive symptoms [26-29]. We found a weak cross-sectional association of anxiety/depression with an increase in heart rate. One might speculate whether increased resting heart rate acts as a mediator of the association between emotional stress and cardiovascular events, but this has not been established [27]. Regarding the third aim of this study, we found, however, no evidence for a mediating effect of heart rate on the association between anxiety/depression and decrease in blood pressure.

This study provides the first data suggesting that the pathophysiological mechanisms underlying the observed association do not involve alteration in heart rate. In recent years, authors of other epidemiological studies have proposed various mechanisms, including the use of antihypertensive medication, but no explanation for the association has been found [8]. Neither did our results explain the relationship between anxiety/depression and blood pressure: First, the association persisted after excluding individuals using antidepressants or antihypertensive medications, thus leaving out any pharmacological explanation. Second, several risk factors usually associated with blood pressure explained very little of the association. Previously, we have discussed that neuropeptide Y and related peptides might be involved in the association between anxiety/depression and decrease in blood pressure [6,11]. These peptides seem associated with sympathetic activity, vascular regulation, and several mental symptoms and disorders.

A recent review deals with other possible mechanisms between chronic psychological states like anxiety and depression and the cardiovascular system [30]. These mechanisms involve changes in sympathetic-parasympathetic balance and the tone of the hypothalamic-pituitary-adrenal axis. Previous findings indicated hypersecretion of cortisol in patients with major depression and various dysregulation of cortisol in other forms of chronic psychological stress, including anxiety and post-traumatic stress disorder. However, dysregulation does not always present with high cortisol levels. It may present with a blunted amplitude of cortisol secretion and impaired responsiveness to acute stressors [30]. As we had no data on stress hormone concentrations, the present study shed no new light on whether dysregulation of cortisol secretion explains part of the association of chronic elevated symptom levels of anxiety/depression with decrease in blood pressure.

Limitations of the present study include that different methods of measuring blood pressure, anxiety and depression were used at baseline compared to at years 11 and 22. We have previously examined and discussed this potential limitation [6,11]. However, our cross-sectional and longitudinal findings in the same population indicated no major problems applying different methods. Additionally, our present findings based on identical methods used at years 11 and 22 (Table 3) confirmed the effect of anxiety and depression on decrease in blood pressure. This makes it even more unlikely that different methods of measuring anxiety/depression and blood pressure could influence the associations we have observed.

Second, it might be argued that measurement of blood pressure and mental symptoms at three single time points gives only snapshots of the participants' situation during 22 year of follow-up. However, the procedures at each wave were standardised and all participants underwent similar examinations. Moreover, in large epidemiological studies like the HUNT study, screening instruments are the standard. Certainly there have been fluctuations in mental symptoms and blood pressure between the three assessments, but it is unlikely that this would bias the association in any particular direction. Statistically, reduced validity of measuring blood pressure or anxiety/depression would weaken rather than strengthen any association between them. It is also of note that the association between a high anxiety/depression symptom level and decrease in blood pressure from baseline to year 22, and from year 11 to 22, was similar to the association we previously found from baseline to year 11 in the same individuals. Given our large data-set based on standardised procedures, we regard that our findings indicate a true association between anxiety/depression and decrease in blood pressure.

Third, we had data only on resting heart rate but not on heart rate variability which is a more precise index of autonomic nervous function than heart rate alone. Low heart rate variability reflects excessive sympathetic and/or inadequate parasympathetic modulation of heart rate and is found associated with depression [26] and anxiety [31] both in general populations [27-29] and in patients with coronary heart disease [26,32]. These findings may explain some of the cardiovascular risk associated with depression [26]. Additionally, a large population-based study has reported that low heart rate variability was associated with the development of hypertension [33]. It follows that adjustment for heart rate variability in analyses of the association between anxiety/depression and blood pressure would be of interest.

## Conclusions

This study benefits from being a large general population-based study covering a wide age range, using repeated assessments of anxiety, depression and blood pressure over 22 years. We found that anxiety and depression were associated with decrease in blood pressure, particularly in individuals with a persistent high symptom level. Heart rate did not mediate the association. The significance of our findings lies partially in stimulating further research on the underlying biological pathways between mental symptoms and blood pressure regulation. Our findings should also have consequences for clinical studies on pharmacological treatment of blood pressure which should take common mental symptoms of participants into account.

## List of abbreviations

HUNT: the Nord-Trøndelag Health study; ADI-12 Index: a one-dimensional anxiety and depression symptom index used at HUNT 1; HADS: the Hospital Anxiety and Depression Scale.

## Competing interests

The authors declare that they have no competing interests.

## Authors' contributions

JH is principal investigator of the HUNT study, and was in charge of the data collection. All authors participated in the study design, evaluation of data and contributed later with text revisions and table revisions. BH planned the study, performed the analyses and drafted the manuscript. UR supervised the statistical analyses. All authors read and approved the final manuscript.

## Pre-publication history

The pre-publication history for this paper can be accessed here:

http://www.biomedcentral.com/1471-2458/11/601/prepub
